# Simultaneous Pleural and Pericardial Effusion as First Clinical Manifestations of Giant Cell Arteritis: A Case Report

**DOI:** 10.7759/cureus.10163

**Published:** 2020-08-31

**Authors:** Vasiliki E Georgakopoulou, Dimitrios Mermigkis, Ourania Kairi, Anna Garmpi, Xanthi Tsiafaki

**Affiliations:** 1 Pulmonology Department, Laiko General Hospital, Athens, GRC; 2 1st Pulmonology Department, Sismanogleio Hospital, Athens, GRC; 3 Intensive Care Unit, Sismanogleio Hospital, Athens, GRC; 4 First Department of Propedeutic Internal Medicine, Laiko General Hospital, National and Kapodistrian University of Athens, Athens, GRC

**Keywords:** temporal arteritis, giant cell arteritis, pleural effusion, pericardial effusion

## Abstract

Giant cell arteritis (GCA) is a chronic granulomatous vasculitis of unknown aetiology occurring in the older patients and affecting mostly the cranial branches of the arteries originating from the aortic arch. GCA is associated with polymyalgia rheumatica (PMR). Clinical features of the disorder include headache, scalp tenderness, jaw claudication, temporal artery abnormalities on physical examination, vision changes, and symptoms associated to PMR. Systemic manifestations include fever, anorexia and weight loss while less rare manifestations are related to the nervous system, the respiratory system, the pericardium and extra-cranial large vessels. Here we report a rare case of simultaneous pleural and pericardial effusion as the first manifestations of GCA. The diagnosis was made with a temporal artery biopsy. Such a diagnosis should, therefore, be considered in older patients presenting with pleuropericardial manifestations, even in the absence of typical clinical features.

## Introduction

Giant cell arteritis (GCA), also called temporal arteritis or cranial arteritis or Horton's disease, is a chronic granulomatous vasculitis of unknown aetiology occurring in older patients. It affects the cranial branches of the arteries originating from the aortic arch. In 10%-15% of cases, the extra-cranial branches of the aortic arch are affected. GCA is related to polymyalgia rheumatica (PMR). Clinical manifestations of the disease include headache, scalp tenderness, jaw claudication, temporal artery abnormalities on clinical examination, vision changes and associated PMR represent the most frequent features of the disease. Systemic symptoms include fever, anorexia and weight loss while less frequent manifestations are related to the central or peripheral nervous system, the respiratory system, the pericardium, and extra-cranial large-vessel involvement [1].

The European League Against Rheumatism (EULAR) guidelines recommend vascular ultrasound as the first diagnostic procedure given adequate expertise and equipment, and in case of unavailability, temporal artery biopsy is the next test for the diagnosis. Corticosteroids remain the first-line therapy for the disease [2].

As mentioned above, pulmonary manifestations of GCA are rare and include in situ pulmonary artery thrombosis and pulmonary infarction, idiopathic isolated pulmonary GCA, pulmonary nodules, interstitial infiltrates, lymphocytic alveolitis, alveolar hemorrhage, and pleural effusion which is typically unilateral, exudative, lymphocyte predominant fluid [3]. Pericardial involvement is also a non-frequent manifestation [3]. We report a case of pleural and pericardial effusion as the first manifestations of GCA.

## Case presentation

An 80-year-old female, non-smoker patient, was admitted to our pulmonology department for fever, dry cough, and shortness of breath over the last four days. She had a history of hypertension, dyslipidemia, appendectomy, and surgically resected pilonidal cyst.

**Figure 1:**
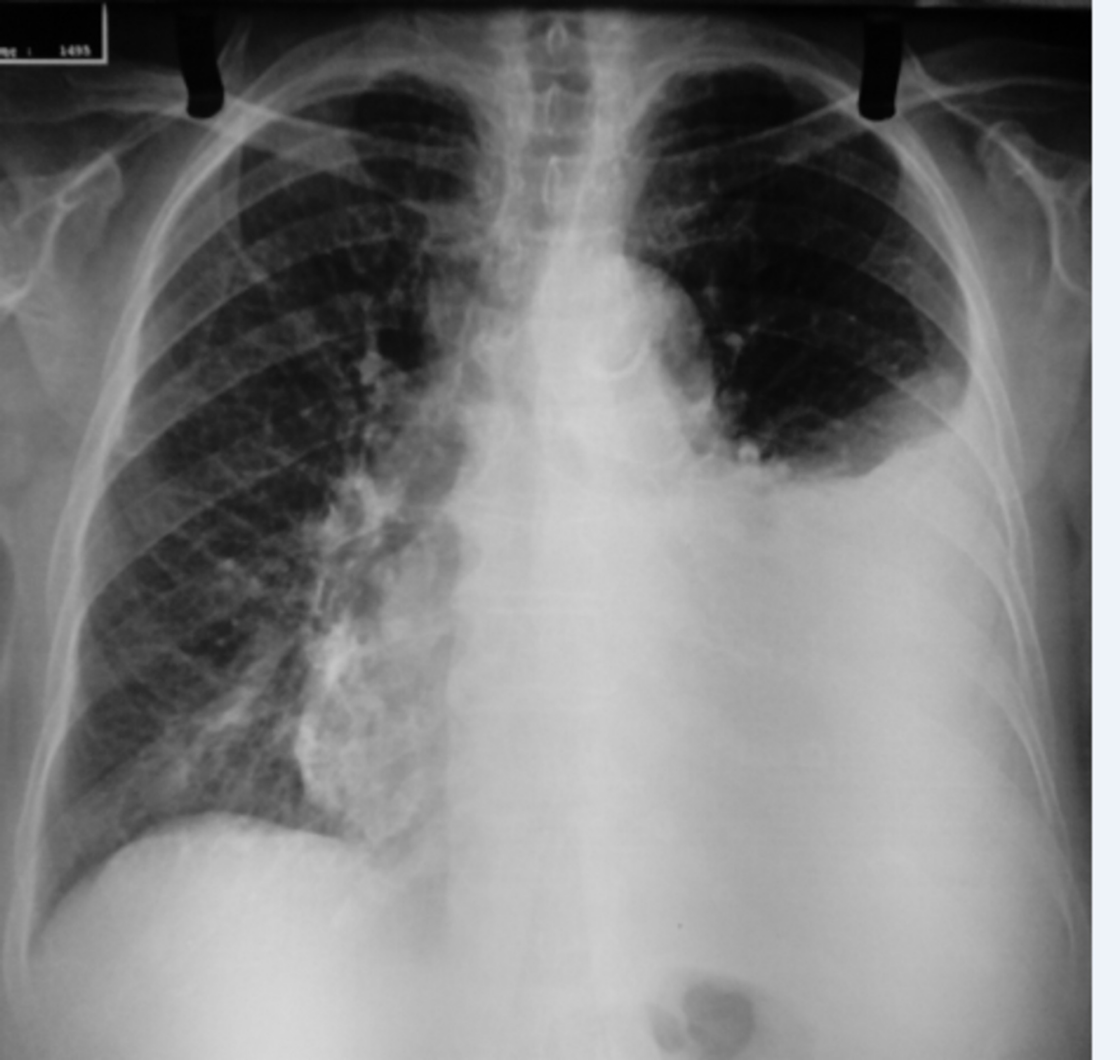
Chest X-ray on admission showing a large left pleural effusion

Clinical examination revealed a febrile patient with decreased breath sounds and dullness on percussion at the base of the left lung. Blood pressure was 140/80 mmHg, heart rate was 97 beats per minute, oxygen saturation was 96% on room air and body temperature 38.8 ° C without abnormal findings on electrocardiography on admission. Chest X-ray showed a large left pleural effusion (Figure 1). Laboratory findings included hemoglobin (Hb) 10.5 g/dL (normal 12-15 g/dL), white blood cells (WBC) 14.31 x 103 /μL (normal 4-11 x 103 /μL), platelets (PTLS) 439 x 103 /μL (normal 150-400 x 103 /μL), erythrocyte sedimentation rate (ESR) 59 mm/hr (normal <20 mm/h) and C-reactive protein (CRP) 101.9 mg/L (normal <6 mg/L). Urinalysis and other blood biochemistry parameters and thyroid-stimulating hormone (TSH) were normal, with the exception of a slightly elevated serum lactate dehydrogenase (LDH) 234 U/L (normal <225 U/L) Table 1.

**Table 1:** Laboratory data on admission AST: aspartate aminotransferase, ALT: alanine transaminase, GGT: gamma-glutamyl transferase, ALP: alkaline phosphatase, LDH: lactate dehydrogenase, CRP: C-reactive protein, ESR: erythrocyte sedimentation rate, Ht: hematocrit, Hb: hemoglobin, PTLS: platelets, WBC: white blood cells

Serum parameters	Patients data
Urea (10-50 mg/dL)	37
Creatinin (0.5-1.5 mg/dL)	0.6
Glucose (60-100 mg/dL)	98
Na (135-148 mEq/L)	137
K (3.5-5.3 mEq/L)	4.3
AST (5-45 U/L)	12
ALT (5-45 U/L)	26
GGT (5-45 U/L)	35
ALP (42-128 U/L)	121
LDH (135-225 U/L)	234
Albumin (3.5-5.1 g/dL)	3.7
Proteins (6.5-8.5 g/dL)	6.5
CRP ( <6 mg/L)	101.9
ESR (0-20mm/h)	59
Ht (37-45%)	33.1
Hb (12-15 g/L)	10.5
PTLS (150-400 x10^3.^μ/L)	439 x 10^3^
WBC (4-11 x10^3^ μ/L)	14.31 x 10^3^

**Figure 2:**
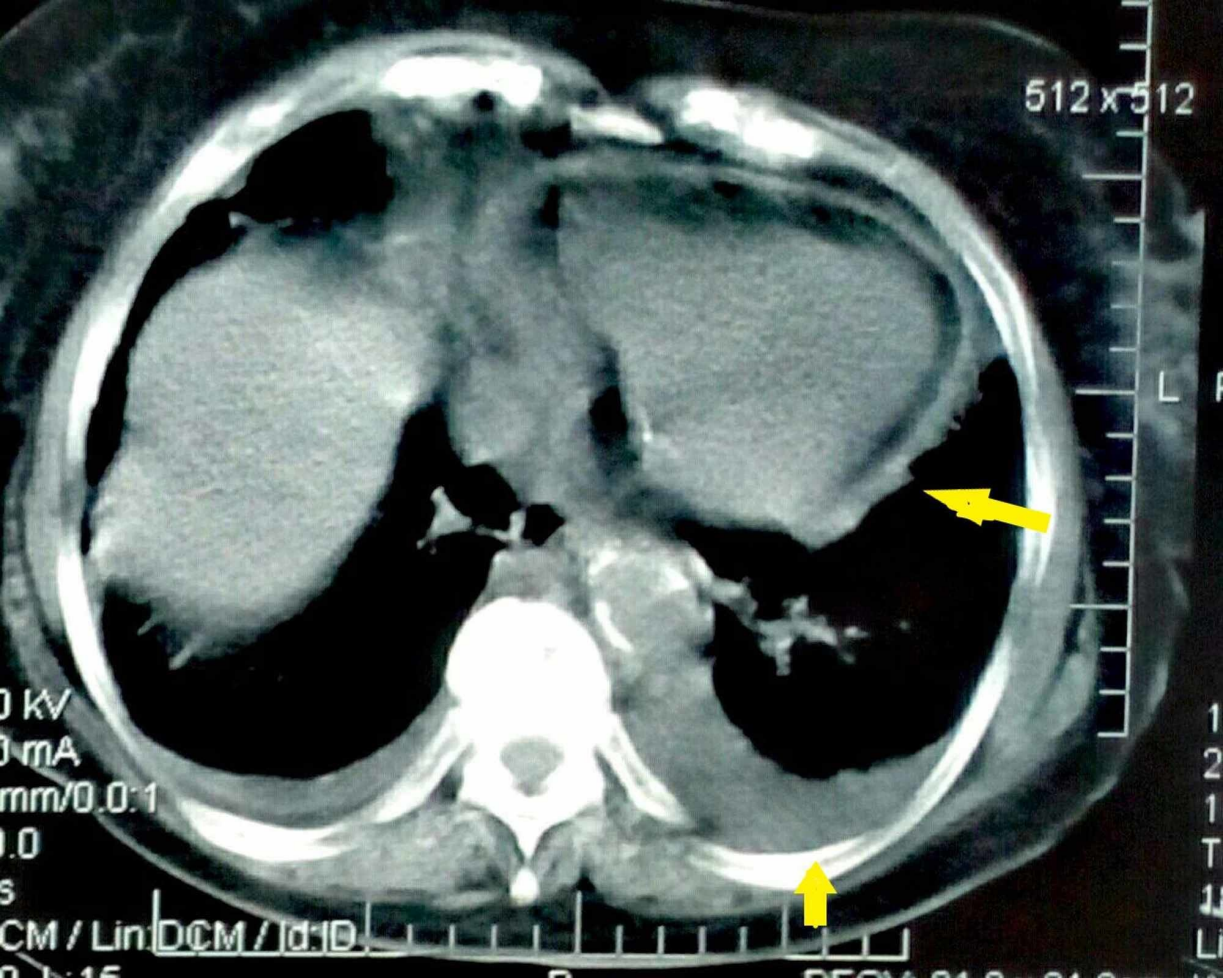
CT of the chest on admission showing a large left pleural effusion, small right pleural effusion, and small pericardial effusion

The patient underwent CT of the chest and CT pulmonary angiogram negative for pulmonary embolism. The chest CT showed large pleural effusion on the left and minimal fluid on the right and minimal pericardial effusion (Figure 2). In addition, the patient underwent echocardiography with normal ejection fraction and valve function and with the presence of small pericardial effusion in the posterior pericardial sac. Thoracentesis revealed a lymphocytic pleural exudate with normal adenosine deaminase (ADA), normal cytology, and negative stains and cultures (Table 2). Autoantibody screening including anti-nuclear antibodies (ANA), rheumatoid factor (RF), anti-cyclic citrullinated peptide antibody (anti-CCP), anti-neutrophil cytoplasmic antibodies (ANCA), and all extractable nuclear antigens (ENA) antibodies (anti-Ro, anti-La, anti-Sm, anti-Jo-1, anti-Mi-2, anti-U1 RNP, anti-Scl-70), was negative. The patient was treated empirically with ceftriaxone and clarithromycin without improvement. Fever persisted, accompanied by an increase in the amount of pleural effusions and pericardial effusion. Tests showed further increases in ESR (100 mm/hr), CRP (178.5 mg/dL) and WBC (15.53 x 103 /μL), while Hb and albumin decreased (9.7 g/dL and 3.0 g/dL, respectively). Cultures and extensive serological tests were negative, as were fundoscopy, abdominal CT, and gastrointestinal endoscopy.

**Table 2:** Characteristics of the pleural fluid LDH: lactate dehydrogenase, ADA: adenosine deaminase

Pleural fluid parameters	Patients data
Glucose (mg/dL)	137.6
LDH (U/L)	135
Proteins (g/L)	3.7
Albumin (g/L)	2.3
ADA (<40 U/L)	9.9
Lymphocytes (% of total fluid cells)	90
Neutrophils (% of total fluid cells)	10

The patient underwent bronchoscopy and pleural biopsy of the left lung. She did not undergo medical thoracoscopy due to bilateral pleural effusions and due to advanced age of the patient, pleural biopsy with Abrams needle was decided to be performed initially. Bronchoscopy with a fiberoptic bronchoscope was performed without obvious lesions and bronchial washings and bronchoalveolar lavage were obtained from lingula and lower left lobe. Cytological and microbiological examination of bronchial washing and bronchoalveolar lavage was negative. Pleural biopsy of the left lung with Abrams pleural biopsy needle was performed and pathological examination revealed non-specific pleuritis.

A more detailed physical examination showed weakness of left temporal artery pulsation. However, the patient did not mention headaches, vision changes, hearing disorders, jaw claudication, or arthralgias. As a final diagnostic, a temporal artery biopsy was conducted revealing temporal arteritis.

**Figure 3:**
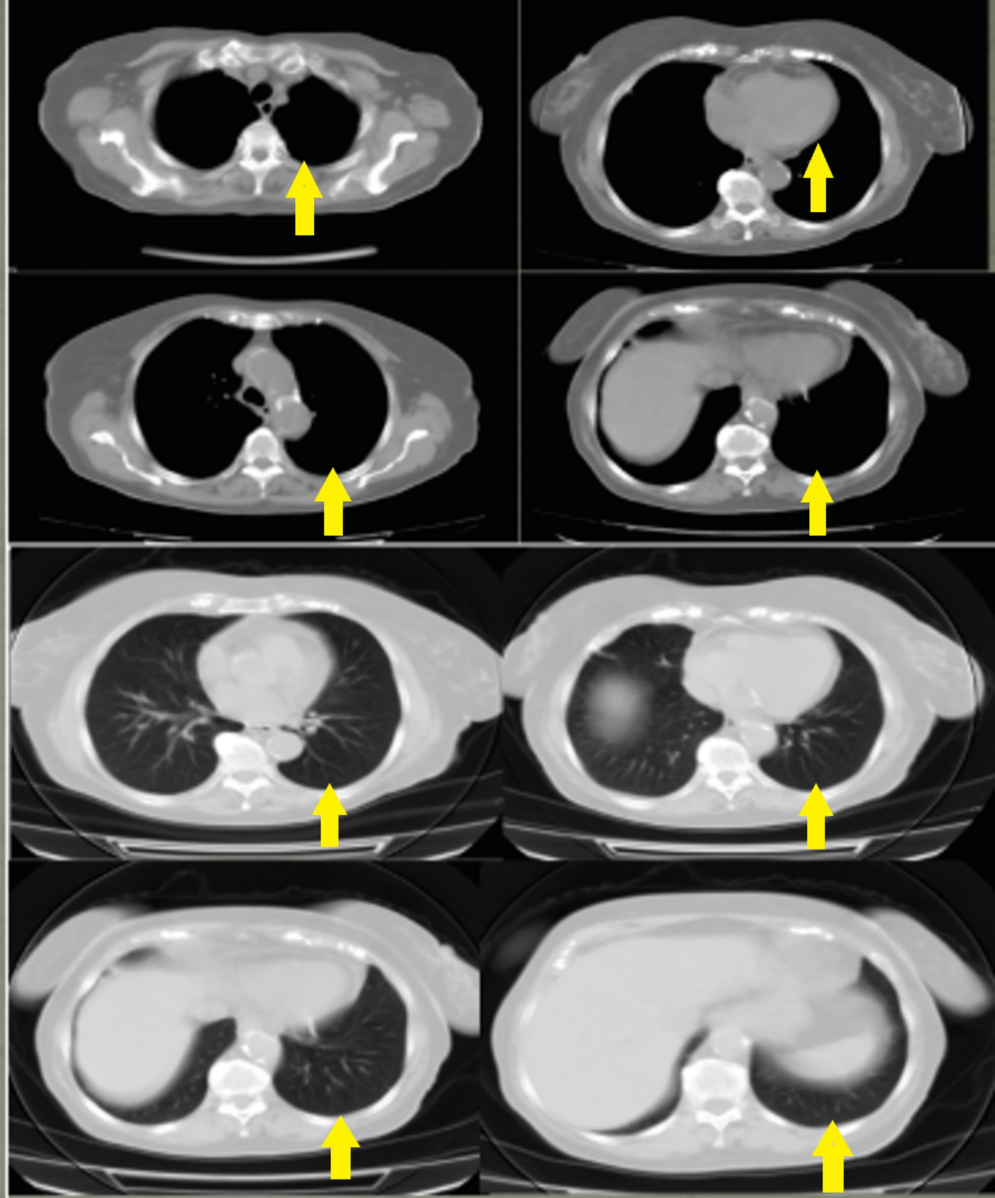
CT of the chest one month after therapy initiation Complete recovery from pleural and pericardial effusions.

The patient received treatment with prednisone (1 mg/Kg/day) which led to complete recovery from fever, pleural and pericardial effusions (Figure 3).

## Discussion

This is a rare case of concurrent pleural and pericardial effusion as the first manifestations of GCA. Pulmonary involvement, and especially pleural involvement, is considered to be rare in GCA, usually occurring in the course of the disease [4]. Romero S et al. reported two patients with temporal arteritis who presented with pleural effusion. They both had typical symptoms of the disease such a headache, fatigue, and jaw claudication. Their pleural fluid was an exudate that responded to treatment with prednisolone [5]. Karachalios et al. described a case of a 73-year-old woman with temporal headache, low-grade fever, fatigue, pleural pain and a left pleural effusion, in whom the diagnosis of temporal arteritis was made with complete recovery after the initiation of corticosteroids [6]. Garcia-Alfranca et al. mentioned a case of a 69-year-old with progressive weakness of her lower extremities, headache, pleural effusion and a biopsy of temporal artery showing lymphocytic and mononuclear infiltrates of the vessel wall, consistent with GCA [7].

In all aforementioned cases, patients had symptoms compatible with the diagnosis of GCA. Few cases, in which pleural effusion was the first manifestation without other symptoms of the disease have been described. Gur et al. reported a 67-year-old woman with biopsy-proven temporal arteritis, who was admitted to the hospital with fever, weight loss, cough, and pleural effusion [8]. Marie et al. mentioned a case of a patient who developed left pleural effusion revealing GCA with rapid improvement, regression of pulmonary clinical symptoms, and complete clearance of pleural effusion, after initiation of steroid therapy, without complaining of typical clinical features of temporal arteritis [4]. Schattner et al. described a case of a 75-year-old man who presented with fever and a small left pleural effusion with a final diagnosis of GCA from temporal artery biopsy and who denied headaches, jaw claudication, or PMR symptoms [9].

Pericardial involvement is an additional rare manifestation of GCA. It was reported for the first time in 1972, when Miller JP reported a 55-year-old-woman who presented with intermittent frontal headaches, night sweats, and episodes of palpitations with pericardial effusion in whom temporary artery biopsy revealed GCA [10]. Tasliyurt et al. described a 74-year-old female who was admitted with the complaints of fatigue, loss of appetite, weight loss, fever, and one-sided headache claudication; echocardiography pericardial effusion was detected. Temporal artery biopsy was conducted confirming GCA [11]. Matsue et al. and Morvai-Illés et al. described a case of a 72-year-old woman and a case of a 68-year-old woman with no fever, nocturnal sweating, headache, or any other symptoms compatible with GCA, and with pericarditis as the first manifestation of the disease, respectively [12,13]. Zenone et al., in their retrospective study, described the prevalence and characteristics of pericardial effusion in patients with GCA. Pericardial effusion was found in four patients of which, three cases had asymptomatic pericardial effusion as initial presentation [14]. Fayyaz et al. performed a systematic review of the literature in order to summarize the epidemiological and clinicopathological aspects of the association between GCA and pericardial effusion and concluded that in 37% of the patients, the pericardial event was the initial disease manifestation and that patients with pericardial involvement represent a clinical phenotype of GCA, different from the cranial or large-vessel forms [15].

Although pericardial and pleural involvement have been reported in patients with GCA, simultaneous occurrence is rare [16]. Valstar et al. reported a 69-year-old woman who presented with malaise, progressive dyspnea and chest pain on inspiration and the diagnosis was pleuropericarditis due to GCA [17]. Cruz et al. reported a 75-year-old male who came to the emergency room due to sudden amaurosis in his left eye, temporal headache, weight loss, low-grade and thoracic pain with a final diagnosis of GCA pleuropericardial involvement [18]. In the same case report, it is mentioned that in a search of the databases MEDLINE and IME (1970-1999), 14 cases of pericardial effusion associated with GCA were found and in half of the cases, a clinically asymptomatic pleural effusion was present [18].

## Conclusions

Simultaneous pleural and pericardial effusions are rarely the first clinical presentation of GCA. Diagnosis of the disease is challenging in these cases, especially if the patient denies typical symptoms consistent with the disease. Such a diagnosis should, therefore, be considered in older patients presenting with pleuropericardial manifestations, even in the absence of typical clinical features.
